# Association between Traffic-Related Air Pollution, Subclinical Inflammation and Impaired Glucose Metabolism: Results from the SALIA Study

**DOI:** 10.1371/journal.pone.0083042

**Published:** 2013-12-10

**Authors:** Tom Teichert, Mohammad Vossoughi, Andrea Vierkötter, Dorothea Sugiri, Tamara Schikowski, Thomas Schulte, Michael Roden, Christian Luckhaus, Christian Herder, Ursula Krämer

**Affiliations:** 1 Institute of Clinical Diabetology, German Diabetes Center, Leibniz Center for Diabetes Research at Heinrich Heine University Düsseldorf, Düsseldorf, Germany; 2 IUF – Leibniz Research Institute for Environmental Medicine at Heinrich Heine University Düsseldorf, Düsseldorf, Germany; 3 Swiss Tropical and Public Health Institute, Basel, Switzerland; 4 University of Basel, Basel, Switzerland; 5 Department of Psychiatry and Psychotherapy, Medical Faculty, Heinrich Heine University Düsseldorf, Düsseldorf, Germany; 6 Department of Endocrinology and Diabetology, University Hospital Düsseldorf, Heinrich Heine University, Düsseldorf, Germany; 7 German Center for Diabetes Research (DZD e.V.), Düsseldorf, Germany; University of Verona, Ospedale Civile Maggiore, Italy

## Abstract

**Background:**

Environmental and lifestyle factors regulate the expression and release of immune mediators. It has been hypothesised that ambient air pollution may be such an external factor and that the association between air pollution and impaired glucose metabolism may be attributable to inflammatory processes. Therefore, we assessed the associations between air pollution, circulating immune mediators and impaired glucose metabolism.

**Methods:**

We analysed concentrations of 14 pro- and anti-inflammatory immune mediators as well as fasting glucose and insulin levels in plasma of 363 women from the Study on the influence of Air pollution on Lung function, Inflammation and Aging (SALIA, Germany). Exposure data for a group of pollutants such as nitrogen oxides (NO_2_, NO_x_) and different fractions of particulate matter were available for the participants' residences. We calculated the association between the pollutants and impaired glucose metabolism by multiple regression models.

**Results:**

The study participants had a mean age of 74.1 (SD 2.6) years and 48% showed impaired glucose metabolism based on impaired fasting glucose or previously diagnosed type 2 diabetes. Only long-term exposure NO_2_ and NO_x_ concentrations showed positive associations (NO_2_: OR 1.465, 95% CI 1.049-2.046, NO_x_: OR 1.409, 95% CI 1.010-1.967) per increased interquartile range of NO_2_ (14.65 µg/m^3^) or NO_x_ (43.16 µg/m^3^), respectively, but statistical significance was lost after correction for multiple comparisons. Additional adjustment for circulating immune mediators or the use of anti-inflammatory medication had hardly any impact on the observed ORs.

**Conclusions:**

Our results suggest that exposure to nitrogen oxides may contribute to impaired glucose metabolism, but the associations did not reach statistical significance so that further studies with larger sample sizes are required to substantiate our findings. Our data do not preclude a role of inflammatory mechanisms in adipose or other tissues which may not be reflected by immune mediators in plasma.

## Introduction

Air pollution is a major environmental risk factor that contributes to the development of a range of acute and chronic conditions such as pulmonary and cardiovascular diseases [1-4]. Novel data indicate that certain pollutants may also contribute to impaired glucose metabolism and the development of type 2 diabetes (T2D) [5-8]. Several epidemiological studies and animal models support this hypothesis by linking elevated levels of several pollutants, like particulate matter (PM) and nitrogen oxides (NO_x_), to increased diabetes incidence rates [6,8-11] and the development of insulin resistance [12-15]. However, the data are still controversial, because not all studies observed associations between air pollution and risk of T2D [16]. 

Main contributors to air pollution are emissions from traffic and industry. Fuels combust to elementary components and volatile fumes of inorganic agents like nitrogen oxides, sulfur dioxide or organic pollutants [17]. A structural characterisation of all emitted agents is difficult due to the complex composition of the fuels, the reactive potential of the intermediates and the conditions of reaction in the engine or combustion chamber. Physico-chemical reactions also take place after the emission, and particulate matter (PM) can increase their diameter from <0.1 µm (PM_0.1_, ultrafine particles) to up to around 1 µm by aggregation. In contrast, coarse particles (PM coarse) between 2.5 and 10 µm in size can arise from mechanical processes like abrasion or originate from sandstorms, volcanic eruption or pollination [18].

There are two major hypotheses on how PM affects health. First, according to Brown et al. [19] around 50% of the inhaled particles are deposited in the lung and can induce a local immune reaction. Due to the small size of the inhaled particles, they are able to penetrate a large surface of the lung and can enter deeply into the respiratory tract. The particles are recognised as foreign matter, and local immune cells like macrophages are activated. These cells can release proinflammatory cytokines and chemoattractants [19-21]. This inflammation aggravates by continued exposure to air pollution and leads to locally elevated concentrations of proinflammatory cytokines and chemokines in the lung. These immune mediators are thought to ”spill over“ into the circulation and trigger cellular inflammatory responses in different tissues. In line with this hypothesis, increased concentrations of proinflammatory acute-phase proteins, cytokines and chemokines such as high-sensitivity C-reactive protein (hsCRP) [22,23] or interleukin-8 (IL-8) [24] have been observed in individuals with high exposure to ambient air pollution compared with individuals who were less exposed. Second, inhaled particles are able to cross cell membranes because of their small diameter and enter the circulation [25,26]. This may lead to the activation of immune cells in different tissues throughout the body and both local and systemic inflammatory processes. In addition, there is also some evidence that air pollutants may activate pulmonary C fibres which could result in the transmission of an inflammatory signal via the autonomic system [27].

The relevance of proinflammatory mechanisms in the development of T2D has been demonstrated extensively in animal models as well as clinical and epidemiological studies [28]. T2D is preceded not only by increased concentrations of proinflammatory immune mediators in the circulation at 10 or more years before its diagnosis [29-31], but also by an upregulation of anti-inflammatory cytokines which most likely represents a futile attempt to counterregulate immunological and/or metabolic stimuli in this prediabetic period [32-34].

Despite the number of epidemiological studies linking ambient air pollution and T2D, it is still not quite clear which components of air pollution are associated with the development of the disease, which pro- and anti-inflammatory cytokines are induced and whether a spill-over of immune mediators into the circulation explains this risk. Therefore, we collected extensive exposure data within the Study on the influence of Air pollution on Lung function, Inflammation and Aging (SALIA, Germany) and measured a range of biomarkers of subclinical inflammation in plasma samples of the study. Using a cross-sectional study design we aimed (i) to assess which components of air pollution were associated with impaired glucose metabolism; (ii) to characterise the associations between these exposures and biomarkers of subclinical inflammation and (iii) to test the hypothesis that the association between air pollution and impaired glucose metabolism can be explained by inflammatory biomarkers in the circulation. 

## Materials and Methods

### Study design and population

The SALIA cohort study is based on consecutive cross-sectional surveys that were performed between 1985 and 1994 as part of the Environmental Health surveys in North-Rhine Westphalia (West Germany). Seven different study areas from the highly industrialised Ruhr district [Dortmund (1985, 1990), Duisburg (1990), Essen (1990), Gelsenkirchen (1986, 1990) and Herne (1986)] were chosen to represent a range of polluted areas with high traffic load as well as steel and coal industries. Additionally two nearby nonindustrialised towns [Borken (1985, 1986, 1987, 1990, 1993, 1994) and Dülmen (1985, 1986)] were chosen as control areas with lower pollution levels. All women aged 54–55 years who lived in the study region were asked to participate; a total of 4,874 women (70%) participated in the study [35]. Thus, the baseline of the study was drawn from a random population of women aged 54-55 years living in the study area. 

In the present study we used data from a follow-up examination performed between 2008 and 2009. For this follow-up all surviving women who participated in the baseline investigation and who had a lung function measurement at baseline were invited. In total, N=834 surviving women were examined, and fasting blood samples were collected in a subgroup of N=363 women. Written informed consent from all study participants was collected. The study was approved by the ethics committee of the Ruhr University in Bochum (Germany).

The participants filled in an extensive questionnaire which included items on symptoms and diagnoses of respiratory and other chronic diseases. It also contained items on single-room heating with fossil fuels and occupational exposures (dust, gases and fumes; extreme temperatures). Socioeconomic status was stratified into two categories by the maximum period of education achieved by the women (<10 years vs. ≥10 years). Women were also grouped according to their smoking habits as never smokers, passive smokers (home, workplace), past smokers, or current smokers (further categorised by <15, 15–30, and >30 pack-years). Data about exposure to indoor mould as covariable was also collected by the questionnaire.

### Air pollution assessment

We applied Geographic Information System (GIS) technique for the assessment of exposures. Using address coordinates exposure to fine particles, nitrogen oxides and traffic was estimated using three different methods:

First, data from monitoring stations maintained by the State Environment Agency covering the Ruhr area in an 8-km grid were used to reflect broad scale spatial variation in air quality. Five-year means of 2003-2007 at the measurement stations for PM_10_ (PM with diameter ≤10 µm) and NO_2_, which were located nearest to the women’s home addresses, were used in order to assess the air pollution exposure.

Second, we used land-use regression models [36-39] and data from a measurement campaign (2008/2009) gained in the framework of the ESCAPE study (European Study of Cohorts for Air Pollution Effects) for assessment of individual long-term exposure. Concentrations of pollutants were measured at 40 sites for NO_2_ and 20 sites for air-borne PM in the study area based on fourteen-day samples for each season and site. The validated land-use regression models were used to assign estimated NO_2_, PM_10_, PM_2.5_ and filter absorbance of PM_2.5_ (soot) concentrations to each individual’s residential address [39].

In SALIA we already found an association between diabetes incidence and traffic-related exposure at baseline and shortly afterwards [6]. Taking the results of this study into account we anticipated that the incidence of diabetes is no immediate effect of air pollution, but evolves after long-term elevated exposures. Since air pollution in the Ruhr Area declined considerably during the last decades we therefore also assigned individually estimated exposure values which describe exposure 10 to 20 years ago. For backextrapolation we used an established ESCAPE procedure which is described in detail in a manual published online (www.escapeproject.org).

For backextrapolation in time a background reference station of the governmental monitoring system was chosen (‘Dortmund Eving’ from LANUV [Federal Ministry of Food, Agriculture and Consumer Protection]) which covered the period from one year before the first SALIA baseline investigation (1984) to the last day of the ESCAPE measurement campaign (October 15, 2009) and was in spatial relation to the SALIA cohort. The means of PM_10_, NO_2_ and NO_x_ for the ESCAPE measuring period and for the period 12 months before to 12 months after the baseline investigation of each SALIA cohort member were calculated from daily and monthly means of PM_10_, NO_2_ and NO_x_ measured at this governmental reference station.

Backextrapolated concentration values for NO_2_, NO_x_, PM_10_, PM_2.5_, PM_coarse_ and PM_absorbance_ were estimated by the ratio method because PM concentrations proportionally declined over time: For each woman the ratio baseline period/ESCAPE period was multiplied with the concentration of the same substance derived from land-use regression, PM_10_ ratio was also multiplied with the fractions of PM_10_. The procedure is explained in detail in the ESCAPE manuals (www.escapeproject.eu).

### Laboratory measurements

Fasting plasma samples were directly stored after collection at -80° and used for all assays. Plasma insulin was measured by ELISA (Mercodia, Uppsala, Sweden). Intra- and inter-assay coefficients of variation for insulin were 2.6% and 3.0%, respectively. Fasting plasma glucose and hsCRP were determined on a Roche/Hitachi Cobas c 311 analyzer (Basel, Switzerland). Plasma levels of IL-1 receptor antagonist (IL-1ra), IL-6, IL-8, tumour necrosis factor α (TNFα), transforming growth factor β_1_ (TGF-β_1_), total adiponectin, high-molecular-weight (HMW) adiponectin, leptin, soluble E-selectin (sE-selectin) and soluble intracellular adhesion molecule 1 (sICAM-1) were measured using Quantikine (IL-1ra, TGF-β_1_, total adiponectin, HMW adiponectin, leptin, sE-selectin, sICAM-1) or Quantikine HS (IL-6, IL-8, TNFα) ELISA kits (R&D Systems, Wiesbaden, Germany). Plasma IL-18 was quantified with the ELISA kit from MBL (Nagoya, Japan). Plasma concentrations of macrophage chemoattractant protein-1 (MCP-1) and interferon gamma-induced protein 10 (IP-10) were assessed using the Human Obesity Base Kit and the Human Cytokine Custom Premix Kit A, respectively (R&D Systems). Bead-based assays were performed using the Bio-Plex 200 System controlled by Bio-Rad Bio-Plex Manager Software 6.0 from Bio-Rad Laboratories (Hercules, CA).

### Statistical Analysis

Descriptive statistics of population characteristics were performed stratified by presence of impaired glucose metabolism (IGM). IGM was defined using the ADA criteria for impaired fasting glucose [40] or physician-diagnosed diabetes. Women with a fasting glucose level below 100 mg/dl or without a previous diagnosis of type 2 diabetes were assumed to have normal glucose metabolism. Women who showed or exceeded the 100 mg/dl glucose limit or were previously diagnosed with T2D belonged to the case population. Continuous variables of inflammation were skewed and therefore presented by median and 25^th^/75^th^ percentiles. We further performed Fisher’s exact test for categorical variables and Wilcoxon test for continuous variables to test for differences between the groups with and without IGM.

Prior regression analysis we controlled the collected data for normal distribution. Continuous variables of inflammation were log-normally distributed and were transformed by the logarithm to the base of 2. After performing multiple linear regression and back-transformation of the regression coefficients, mean ratios (MR) and 95% confidence intervals (CI) were presented. MR describe the relative change of the biomarker when the air pollution exposure marker is increased by one unit. As unit we chose the respective interquartile range (IQR), which is the difference between the 75^th^ quartile and the 25^th^ quartile of the distribution of the particle pollution variables.

Logistic regression was used to analyse the association between air pollution exposure and the presence of IGM as well as the association between biomarkers of inflammation and the presence of IGM. Odds ratios with corresponding 95% CI indicate the chance of having IGM if exposure to air pollution is increased by one IQR or if the concentration of log_2_-transformed inflammatory markers increase by one unit. 

All P values are two-sided. P values <0.05 were considered to indicate nominal statistical significance without correction for multiple testing. In order to correct for multiple testing, we adjusted the significance level in each analysis according to Bonferroni (α = 0.05 divided by the number of comparisons). All statistical analyses were performed using SAS version 9.3 (SAS Institute, Cary, NC).

## Results

### Characteristics of the study population

In our study population of 360 women, 174 (48.3%) had IGM as defined by impaired fasting glucose (78.7% of women with IGM) or a previous diagnosis of T2D by their physicians (21.3% of women with IGM). Women with IGM had a higher BMI, higher fasting glucose and insulin concentrations than women without IGM, but did not differ with regard to age, education, smoking status, place of residence (urban vs rural) or exposure to indoor mould (Table 1).

**Table 1:** Characteristics of the SALIA population stratified by impaired glucose metabolism (IGM).

Variable	Impaired glucose metabolism	Normal glucose metabolism	P
n	174	186	-
Age [years]	74.2 ± 2.7	73.9 ± 2.5	0.4065
BMI [kg/m^2^]	29.2 ± 4.7	26.1 ± 4.1	<0.0001
<10 years education [%]	35.3	31.9	0.5237
Smoking (current/former/never) [%]	2.9/14.4/82.8	3.2/17.7/79.0	0.6817
Passive smoking [%]	54.6	61.3	0.2020
Exposure to indoor mould [%]	12.6	13.4	0.8763
Urban residence [%]	54.0	52.7	0.8329
Fasting glucose [mg/dl] *	110.5 ± 12.0	91.2 ± 6.0	<0.0001
Fasting insulin [µU/ml] *	8.6 ± 3.9	6.2 ± 3.6	<0.0001
HOMA-IR *	2.4 ± 1.2	1.4 ± 0.9	<0.0001
Previous diagnosis of T2D [%]	21.3	-	<0.0001

Data are given as mean ± SD or percentages.

^*^ Based on 137 women without previous diagnosis of T2D in the IGM group. BMI, body mass index; HOMA-IR, homeostasis model assessment-insulin resistance; T2D, type 2 diabetes.

### Association between biomarkers of subclinical inflammation and IGM

Plasma levels of 14 immune mediators for women with and without IGM are presented with their median concentrations and 25^th^ and 75^th^ percentiles in Table 2. In unadjusted comparisons, we found higher concentrations for the acute-phase protein hsCRP, the proinflammatory cytokine leptin, the anti-inflammatory cytokine IL-1ra and the chemokine MCP-1/CCL2 among women with IGM, whereas plasma levels of total and HMW adiponectin were lower in IGM. There were no differences between women without and with IGM with regard to concentrations of IL-6, IL-18, TNFα, TGF-β1, IP-10/CXCL10, IL-8/CXCL8 and sICAM-1 (all P>0.05). 

**Table 2:** Plasma concentrations of biomarkers of subclinical inflammation stratified by the presence of impaired glucose metabolism (IGM).

Biomarker	Impaired glucose metabolism	Normal glucose metabolism	P*	P**
Acute-phase protein				
hsCRP [mg/dl]	0.3 (0.2; 0.5)	0.2 (0.1; 0.4)	0.0019	0.0008
Proinflammatory cytokines				
IL-6 [pg/ml]	1.6 (1.0; 2.5)	1.4 (0.9; 2.2)	0.2868	0.3113
IL-18 [pg/ml]	250.6 (197.5; 325.6)	237.1 (185.5; 308.0)	0.6578	0.5457
TNFα [pg/ml]	1.4 (1.0; 2.0)	1.4 (1.0; 1.9)	0.8703	0.7904
Leptin [ng/ml]	19.6 (13.6; 27.6)	13.1 (8.0; 20.7)	<0.0001	<0.0001
Antiinflammatory cytokines				
IL-1ra [pg/ml]	268.7 (194.7; 402.7)	210.2 (159.5; 275.6)	<0.0001	<0.0001
TGF-β1 [ng/ml]	5.7 (3.8; 8.2)	5.4 (3.5; 8.3)	0.9053	0.8479
Total adiponectin [µg/ml]	7.9 (5.1; 10.9)	9.5 (6.8; 13.0)	0.0001	<0.0001
HMW adiponectin [µg/ml]	3.8 (2.4; 5.5)	5.1 (3.2; 7.1)	<0.0001	<0.0001
Chemokines				
MCP-1/CCL2 [pg/ml]	136.6 (115.6; 167.9)	131.8 (105.2; 163.3)	0.0108	0.0068
IP-10/CXCL10 [pg/ml]	47.2 (31.9; 71.1)	47.2 (31.5; 68.9)	0.4812	0.4755
IL-8/CXCL8 [pg/ml]	11.9 (9.4; 15.2)	11.3 (9.1; 15.2)	0.1583	0.1242
Soluble adhesion molecules				
sE-selectin [ng/ml]	29.1 (22.0; 39.1)	27.5 (21.1; 32.7)	0.0655	0.0864
sICAM-1 [ng/ml]	210.4 (184.2; 255.1)	218.2 (185.8; 264.6)	0.5342	0.4313

Data are given as median (25^th^ percentile; 75^th^ percentile).

*P values for the unadjusted comparison of both groups; **P values for the comparison of both groups adjusted for age, BMI, smoking status, passive smoking, education, exposure to indoor mould and season of blood sampling. Based on 14 comparisons, the Bonferroni-adjusted P value to indicate statistical significance after correction for multiple testing is 0.05/14 = 0.0036.

**Figure 1:**
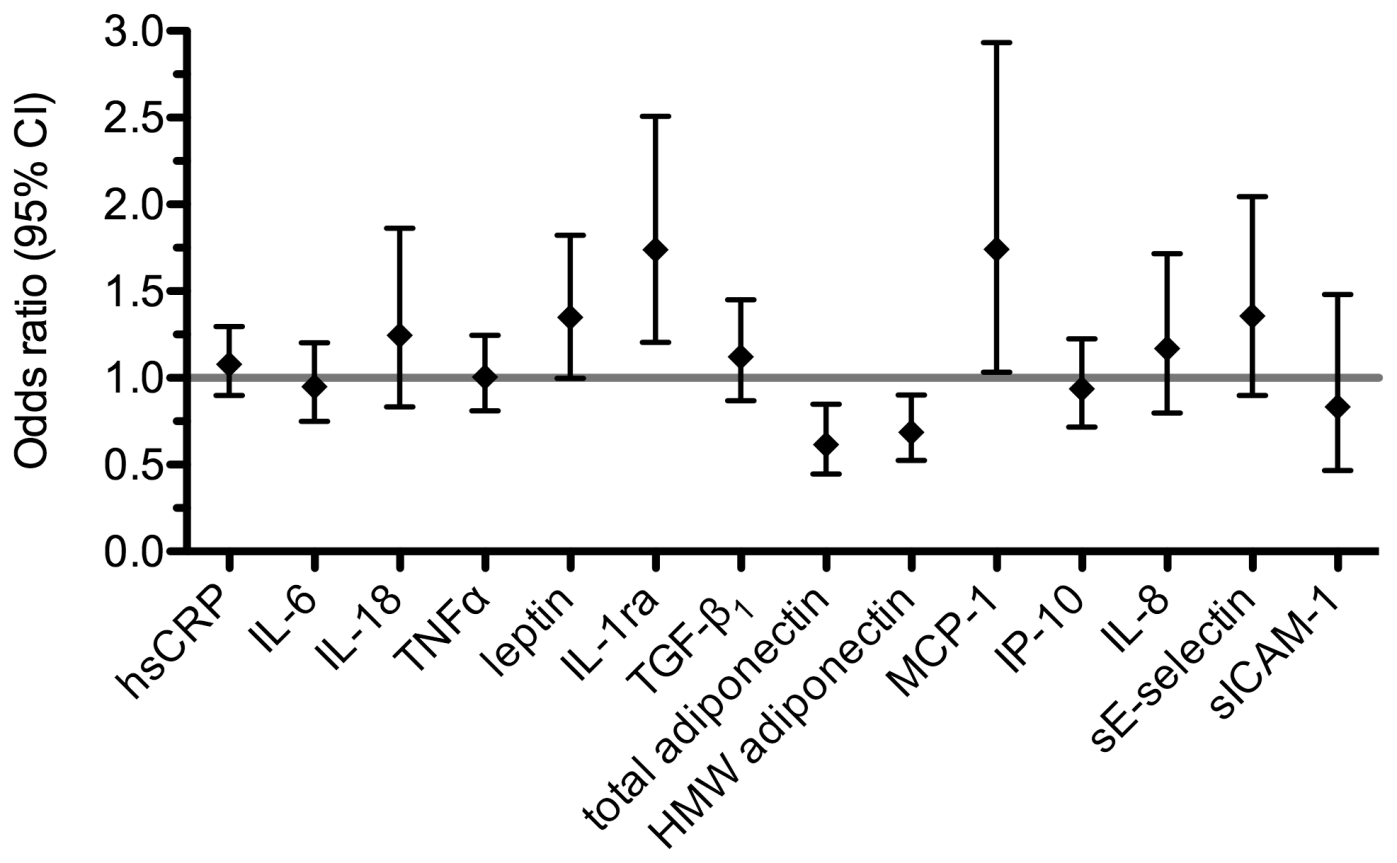
Association between circulating immune mediators and IGM. Odds ratios are adjusted for age, BMI, smoking status, passive smoking, education, exposure to indoor mould and season of blood sampling.

We further analysed the association between circulating immune mediators and IGM by multiple linear regression adjusting for multiple confounders. As shown in Figure 1, high levels of leptin, IL-1ra and MCP-1 were significantly associated with IGM after adjustment for age, BMI, smoking status, passive smoking, education, exposure to indoor mould and season of blood sampling. For total and HMW adiponectin, the inverse association remained significant after adjustment for the aforementioned confounders (Figure 1). However, the association between MCP-1 and IGM was only nominally significant, but not after Bonferroni adjustment for multiple testing.

We also obtained information about the medication of the participants with non-steroidal anti-inflammatory drugs from the questionnaire. Adding this information to the model had virtually no effect on the results (data not shown).

### Exposure to air pollution and IGM

Data for exposure to air pollution were collected from local monitoring stations near the residence of the participants or individually assigned from land-use regression estimates and are summarised in Table 3 stratified by presence of IGM. In the unadjusted analyses, exposure levels did not differ between women without and with IGM. Only back-extrapolated NO_2_ and NO_x_ levels tended to be higher in women with IGM (P=0.16 and P=0.11, respectively). 

**Table 3:** Exposure to traffic-related air pollution stratified by presence of impaired glucose metabolism.

Variable	Impaired glucose metabolism	Normal glucose metabolism	P
NO_2_ * [µg/m^3^]	29.1 ± 8.3	28.5 ± 7.7	0.42
NO_2_^†^ [µg/m^3^]	39.7 ± 10.9	37.8 ± 9.8	0.16
NO_x_ * [µg/m^3^]	47.8 ± 20.0	45.6 ± 19.4	0.21
NO_x_^†^ [µg/m^3^]	74.1 ± 31.2	69.3 ± 30.0	0.11
PM_2.5_ absorbance* [10^-5^/m]	1.5 ± 0.5	1.5 ± 0.5	0.88
PM_2.5_ absorbance**^*†*^** [10^-5^/m]	2.9 ± 0.9	2.8 ± 0.8	0.88
PM_2.5_ * [µg/m^3^]	17.9 ± 1.4	18.0 ± 1.4	0.56
PM_2.5_^†^ [µg/m^3^]	34.0 ± 3.2	34.0 ± 3.1	0.74
PM coarse* [µg/m^3^]	9.6 ± 1.6	9.6 ± 1.8	0.89
PM coarse^†^ [µg/m^3^]	18.2 ± 3.3	18.2 ± 3.3	0.79
PM_10_ * [µg/m^3^]	27.0 ± 2.1	27.1 ± 2.3	0.89
PM_10_^†^ [µg/m^3^]	51.2 ± 4.9	51.0 ± 4.9	0.90

Data represent mean ± SD.

^*^ Measured within the observation period (2008/2009) by local air quality measurement stations and calculated for the participants’ residence by LUR model.

^†^ Back-extrapolated concentrations of exposure variables (see section “Air pollution assessment”).

We also assessed the association between air pollution and IGM by using logistic regression modeling. All exposure variables were positively related to IGM after adjusting for age, BMI, smoking status, passive smoking, indoor mould, years of education and season of blood sampling (Table 4). In particular nitrogen oxides showed a strong association with IGM. The associations between both back-extrapolated NO_2_ (NO_2_^†^) and back-extrapolated NO_x_ (NO_x_^†^) and presence of IGM were nominally statistically significant with an OR of 1.465 (95% CI 1.049–2.046, P=0.0249) and 1.409 (95% CI 1.011-1.967, P=0.0437), respectively. However, it has to be noted that both associations were not significant after Bonferroni adjustment for multiple testing.

**Table 4:** Adjusted odds ratios (OR) and 95% confidence intervals (CI) for the association between components of air pollution and presence of IGM.

		**OR (95% CI)**		
**Pollutant**	**Back-extrapolated variables**	P*	**Variables from the survey 2008/2009**	P*
NO_2_	1.465 (1.049-2.046)	0.0249	1.218 (0.909-1.630)	0.1862
NO_x_	1.409 (1.010-1.967)	0.0437	1.224 (0.926-1.617)	0.1562
PM_2.5_ absorbance	1.258 (0.947-1.670)	0.1127	1.110 (0.889-1.385)	0.3556
PM_2.5_	1.149 (0.812-1.627)	0.4331	1.117 (0.808-1.543)	0.5034
PM coarse	1.128 (0.794-1.603)	0.5004	1.075 (0.833-1.388)	0.5781
PM_10_	1.209 (0.871-1.679)	0.2561	1.145 (0.896-1.465)	0.2794

Odds ratios are standardised to one IQR increase of exposure variables and adjusted for age, BMI, smoking status, passive smoking, education, exposure to indoor mould and season of blood sampling.

^*^ Based on 12 comparisons, the Bonferroni-adjusted P value to indicate statistical significance after correction for multiple testing is 0.05/12 = 0.0042.

### Impact of immune mediators on the association between nitrogen oxides and IGM

**Figure 2:**
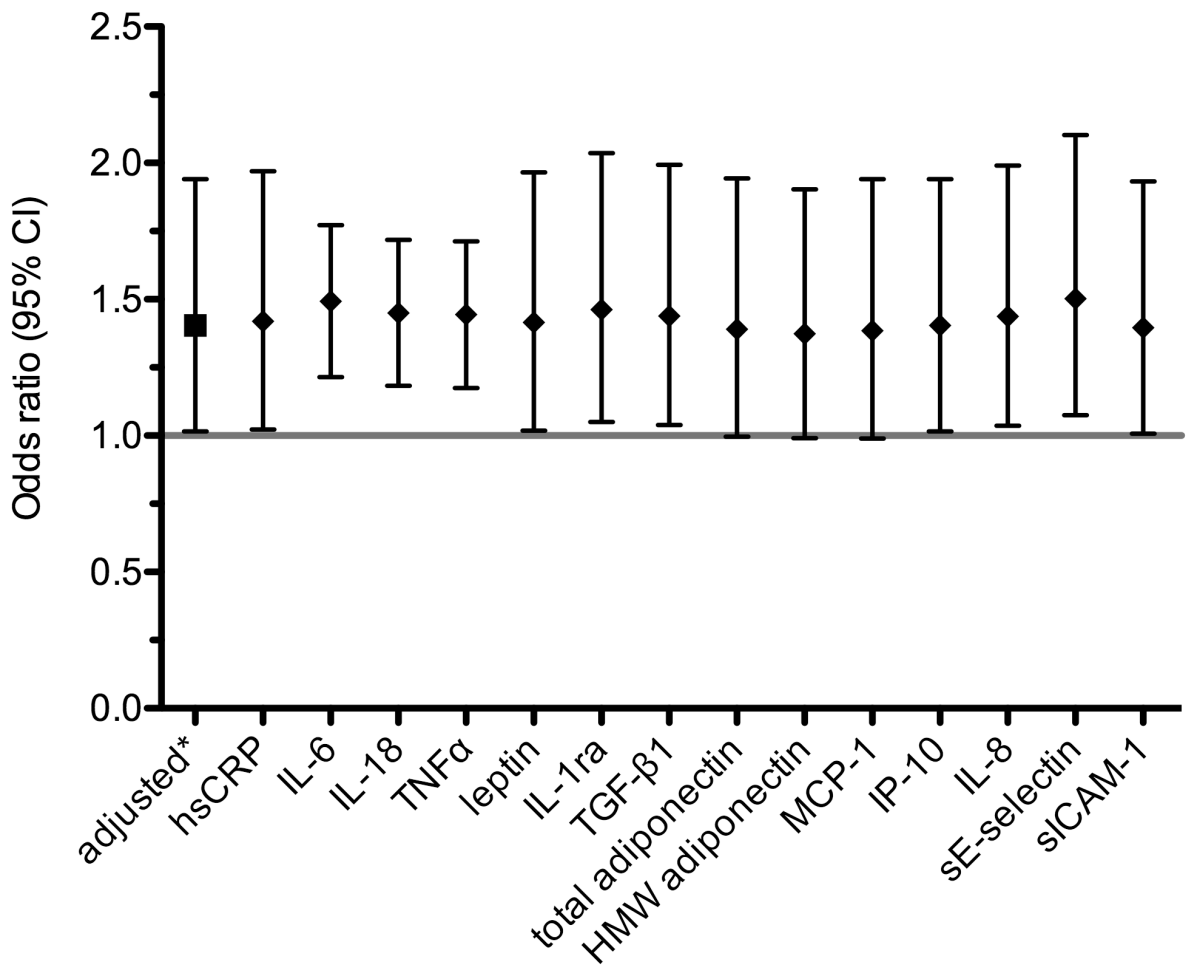
Impact of adjustment for immune mediators on the relationship between IGM and NO_2_^†^. *Adjusted for age, BMI, smoking status, passive smoking, education, exposure to indoor mould and season of blood sampling. All additional models are adjusted for the aforementioned covariables and the immune mediator indicated on the x-axis.

After we examined the association between air pollution and IGM, we tested if the significant association between NO_2_^†^ and IGM was independent of circulating immune mediators. Therefore we extended the adjustment model separately for each of the measured immune mediators. Figure 2 shows how each immune mediator influenced the OR between NO_2_^†^ and IGM. By adjusting the association for the standard set of confounders the OR is given by 1.465 (95% CI 1.049–2.046). Additional adjustment for each of the analysed immune mediators had virtually no effect on the observed OR (Figure 2). Similar results were obtained for the analogous analysis for NO_x_^†^ (Figure S1).

**Figure 3:**
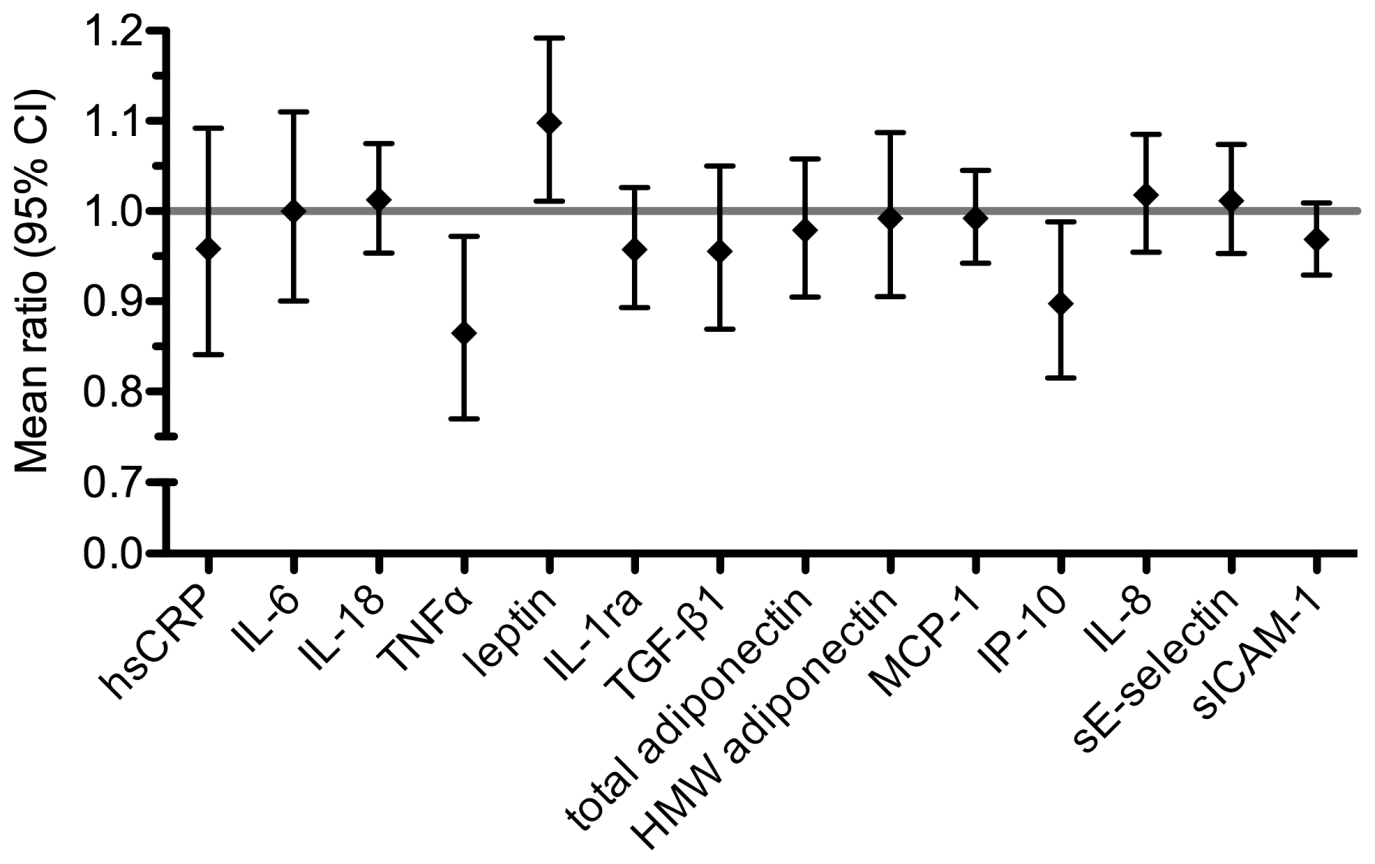
Association between circulating immune mediators and NO_2_^†^. Mean ratios are adjusted for age, BMI, smoking status, passive smoking, education, exposure to indoor mould and season of blood sampling. Ratios represent the relative increase of the particular serological marker concentration by 1-IQR increase of NO_2_^†^ levels (IQR=14.65 µg/m^3^).

In order to better understand why this additional adjustment had hardly any impact on the OR for the association between nitrogen oxides and IGM, we used multiple linear regression and assessed the association between NO_2_^†^ as continuous, independent variable and each immune mediator. As shown in Figure 3, most immune mediators were not significantly associated with NO_2_^†^, and only leptin showed a weak positive association, whereas weak inverse association were observed for TNFα and IP-10/CXCL10. P values for these associations were between 0.01 and 0.05 and thus only nominally significant, but not after correction for multiple testing leading to a significance level of 0.05/14 = 0.0036. Results were similar for NO_x_^†^ (Figure S2).

## Discussion

This cross-sectional study examined the association between ambient air pollution, subclinical inflammation and IGM in elderly women. Our main findings were as follows: (i) Ambient levels of NO_2_ and NO_x_, but not of PM, were nominally significantly associated with IGM, but not after adjustment for multiple comparisons. (ii) The observed effect sizes were independent of plasma levels of biomarkers of subclinical inflammation. (iii) In line with this, we found associations between subclinical inflammation and IGM, whereas associations between nitrogen oxides and subclinical inflammation were less pronounced. 

Several studies demonstrated the influence of air pollution on the prevalence or the prospective risk of T2D or hallmarks of the disease like insulin resistence. One important class of pollutants is the group of nitrogen oxides (NO_x_ and NO_2_). Their concentrations correlate with traffic load which means that their sources are predominantly combustion processes in cars [6,18,41,42]. With respect to adverse health effects NO_2_ and NO_x_ represent commonly studied nitrogen compounds. It was shown that NO_2_ is related to the impairment of glucose metabolism at relatively high [6,9] and low [5,8,43] concentrations. The development of IGM is a continuous and progressive process which can last many years before T2D is manifest or diagnosed. Our prior study in women also based on the SALIA cohort reported a 42% increase in the diabetes incidence rate per IQR of PM or NO_2_ in a study population with a baseline exposure to NO_2_ of 47 µg/m^3^. These results were corroborated by two more recent studies. One study was based on women in the United States. The main finding was a 24% higher incidence rate for diabetes for an increase of ambient NO_2_ concentrations by 11 µg/m^3^ (baseline exposure 43 µg/m^3^) [9]. The second study was established in Denmark and started 1993 with more than 50,000 men and women. Even if the overall NO_2_ concentration (15 µg/m^3^) was lower compared to the German SALIA and American cohorts, the authors confirmed the described association between increased diabetes incidence rates for increasing NO_2_ levels [8]. Our results are in line with these previously published results and strengthen the notion of adverse metabolic health effects of elevated traffic-related nitrogen oxides. NO_2_ has also been examined in the context of diabetes-related mortality rates in a large Danish cohort with the main finding of a strong association between the mortality rate and increasing levels of ambient NO_2_ [43].

A second class of pollutants that has been investigated as potential risk factor comprises PM of different particle sizes. In our study, different exposure measures of PM showed no significant association with IGM. This is in contrast to an Iranian study [13] which examined the influence of lifestyle and environmental factors on the health of children. Their results suggest that exposure to elevated PM_10_ levels contributes to the development of T2D by exacerbating insulin resistance, measured as HOMA-IR. However, it has to be noted that compared to our study, the exposure levels of PM_10_ were much higher (122 ± 44 µg/m^3^) which may explain a more pronounced effect. In a study from Taiwan [44] it was shown that long-term exposure to PM_2.5_ contributed to a dysregulation of glucose metabolism, assessed as elevated levels of HbA1c and fasting glucose. In line with this, a rise of the T2D incidence rate by 1% was observed if the concentration of PM_2.5_ increased by 10 µg/m^3^, even at relatively low levels in a study from the United States [7]. These results are corroborated by a study from Canada, which found an 11% increased T2D incidence rate by a 10 µg/m^3^ increased PM_2.5_ level in a cohort of more than 60,000 participants [11]. Recent reports underline the toxicological potential and importance of PM_2.5_ [4] for a decrease of insulin sensitivity even after sub-acute exposure to low levels of 5-10 µg/m^3^ PM_2.5_ [12]. However, it has to be noted that other studies were not able to confirm the association between nitrogen oxides or PM and IGM [5,16]. These studies were performed in rural areas with comparatively low pollution levels, which may be the cause of the non-significant findings, because lower effect sizes for lower exposure levels require considerably larger cohorts to obtain the same statistical power as a study with larger effect sizes at higher exposure levels.

Although our findings are only nominally significant, but not after adjustment for multiple comparisons, the observed effect sizes are in line with previous reports on the toxicological effect of air pollution and in particular on potential associations between nitrogen oxides and IGM [5,6,8,9,43]. Besides this result there were no consistent associations between air pollution and the dysregulation of biomarkers of subclinical inflammation. We observed inverse assocations between the exposure to air pollutants and the proinflammatory cytokine TNFα and the chemokine IP-10. This might be related to a decreased immune response of the elderly participants, because both mediators are originally released by T_H_1 immune cells. One can speculate that the advanced age of our study population could have diminished the immune response.

The positive and significant association between NO_2_^†^ and circulating leptin could indicate a pulmonary inflammation [45], if those levels are connected to the leptin concentrations in the lung and respond in line with the first hypothesis. The infiltration of the circulating system by solid pollutants shows some parallels to microparticles, derived from endothelial or apoptotic cells, which might accelerate the inflammatory process. Those endogenous remnants increase in vitro the ICAM-1 and VCAM-1 concentration and their mRNA levels, which might be the result of activating the MAP kinase pathway [46]. However, we were not able to detect any positive association between PM_2.5_ or PM_10_ with sICAM-1 levels like former studies [47]. Furthermore, we wanted to study the influence of inflammation on the association between air pollution and IGM. Therefore we added the single inflammatory mediators separately into the model assessing the association between NO_2_^†^ and IGM, but the strength of the assosication remained almost unchanged. 

Previously published reports on the association between air pollutants and systemic inflammation in human study populations were inconsistent. Seaton et al. described an assocation between fine particles like PM_10_ and the acute-phase protein CRP in elderly people [48]. This finding was confirmed by studies in children [10] and elderly men [47]. Rückerl et al. also found a relationship between elevated levels of NO_2_, carbon monoxide and ultrafine particles and increased circulating CRP levels [47]. Moreover, several studies reported associations between PM and the proinflammatory cytokine IL-6 [10,12,20,49]. In contrast, Seaton et al. were not able to detect an effect of PM_10_ on IL-6 plasma concentrations [48], which was also absent in a study in mice by Fonken et al. [50]. Also the association between PM and CRP could not be supported by all studies [23,51].

Besides the male cohort of Seaton et al. [48], all other mentioned cohorts included participants who were younger than in the SALIA study. We cannot exclude a survivor effect in our cohort. This means that it is possible that women who were more susceptible to air pollution and therefore showed a more intensive immune reaction were not included in our study any more, because they mave have died earlier or may have been too ill to participate in the study. An alternative explanation of our null results regarding the role of biomarkers of subclinical inflammation in the link between air pollution and glucose metabolism might be that our elderly participants might have been affected to various degrees by different age-related chronic diseases, which are mostly proinflammatory so that the level of circulating immune mediators may be less affected by exogenous stimuli. 

Our study has several strengths and limitations that should be discussed briefly. This is the first study which investigates the association between air pollution, plasma levels of inflammatory biomarkers and IGM using extensive measures of air pollutants and a detailed immunological phenotyping of study participants. Our results are consistent with previously published studies on the associations between nitrogen oxides and IGM. 

We are limited in the conclusions, because we performed a cross-sectional study. Our sample size was moderate, so that we cannot exlude that we may have missed associations that would have been significant in larger cohorts. We only examined elderly women so that a bias in certain findings due to a survivor effect cannot be excluded. Data regarding the prevalence of gestational diabetes as risk factor for subsequent IGM in later life were not available. Oral glucose tolerance tests were not performed in the study, so women with impaired glucose tolerance who would also have been included in the IGM group could not be diagnosed by their 2-hour glucose levels. Moreover, data for HbA1c were not available so that HbA1c levels could not be included in the definition of IGM. The lack of an effect for PM may partly be due to the fact that twice as many calibration sites were used for NO_2_ than for PM. This may have weakened the ability of the particle model to represent local traffic particles, which decline to urban background within 100 m. The out-of-sample R^2^ of the different models for the Ruhr Area, however, were quite high and comparable (NO_2_: 89%, PM_2.5_: 86%, PM_2.5_ absorbance: 97%, PM_10_: 69%). Thus, differential measurement error from this source might not be an explanation for the difference in effects. Finally, we examined the effects of various exposure measures separately, although everybody is exposed to a mixture of particles. It is conceivable that all components of air pollution have detrimental effects on health in their mixture which argues for the use of more complex regression models to take additive or synergistic effect of various pollutants into account [9]. It should be noted that the systemic inflammatory markers were evaluated at the same point in time as IGM. The latter may have developed after long-term exposure to elevated air pollution during the last decades. In contrast the inflammatory markers might reflect a reaction after a more short-term exposure. The exposure in 2008/2009 was much lower than the exposure at study baseline and may not be associated with systemic inflammatory reactivities any more. 

In conclusion, we provide evidence that air pollution may be linked to IGM with more pronounced associations for nitrogen oxides than for PM. However, it should be noted that our results were not robust against adjustment for multiple comparisons. Therefore, further studies, preferably with larger sample sizes and using a prospective design, will be necessary to clarify to what extent air pollution and IGM are associated. Although we observed some nominally significant associations between between the up- and downregulation of pro- and anti-inflammatory mediators and IGM, plasma levels of these immune mediators did not explain the association between exposure to NO_2_ or NO_x_ and IGM. Our results do not preclude the relevance of local inflammatory processes in adipose tissue or the skeletal muscle which may be triggered by exposure to air pollution and which may contribute to the development of IGM. Therefore, further mechanistic studies are required to unravel to role of immune activation in the association between air pollution and risk of T2D.

## Supporting Information

Figure S1 **Impact of adjustment for immune mediators on the relationship between IGM and NO_x_^†^.** *Adjusted for age, BMI, smoking status, education, exposure to indoor mould and season of blood sampling.
All additional models are adjusted for the aforementioned covariables and the immune mediator indicated on the x-axis.
(TIFF)

Figure S2 **Association between circulating immune mediators and NO_x_^†^.** Mean ratios are adjusted for age, BMI, smoking status, education, exposure to indoor mould and season of blood sampling. Ratios represent the relative increase of the particular serological marker concentration by 1-IQR increase of NO_x_^†^ levels (IQR=43.16 µg/m^3^).
(TIFF)
