# Supplementary figures and images for: Association between Traffic-Related Air Pollution, Subclinical Inflammation and Impaired Glucose Metabolism: Results from the SALIA Study
Source: PLoS One. 2013 Dec 10;8(12):e83042. doi: 10.1371/journal.pone.0083042 (PMC3858363; doi:10.1371/journal.pone.0083042)

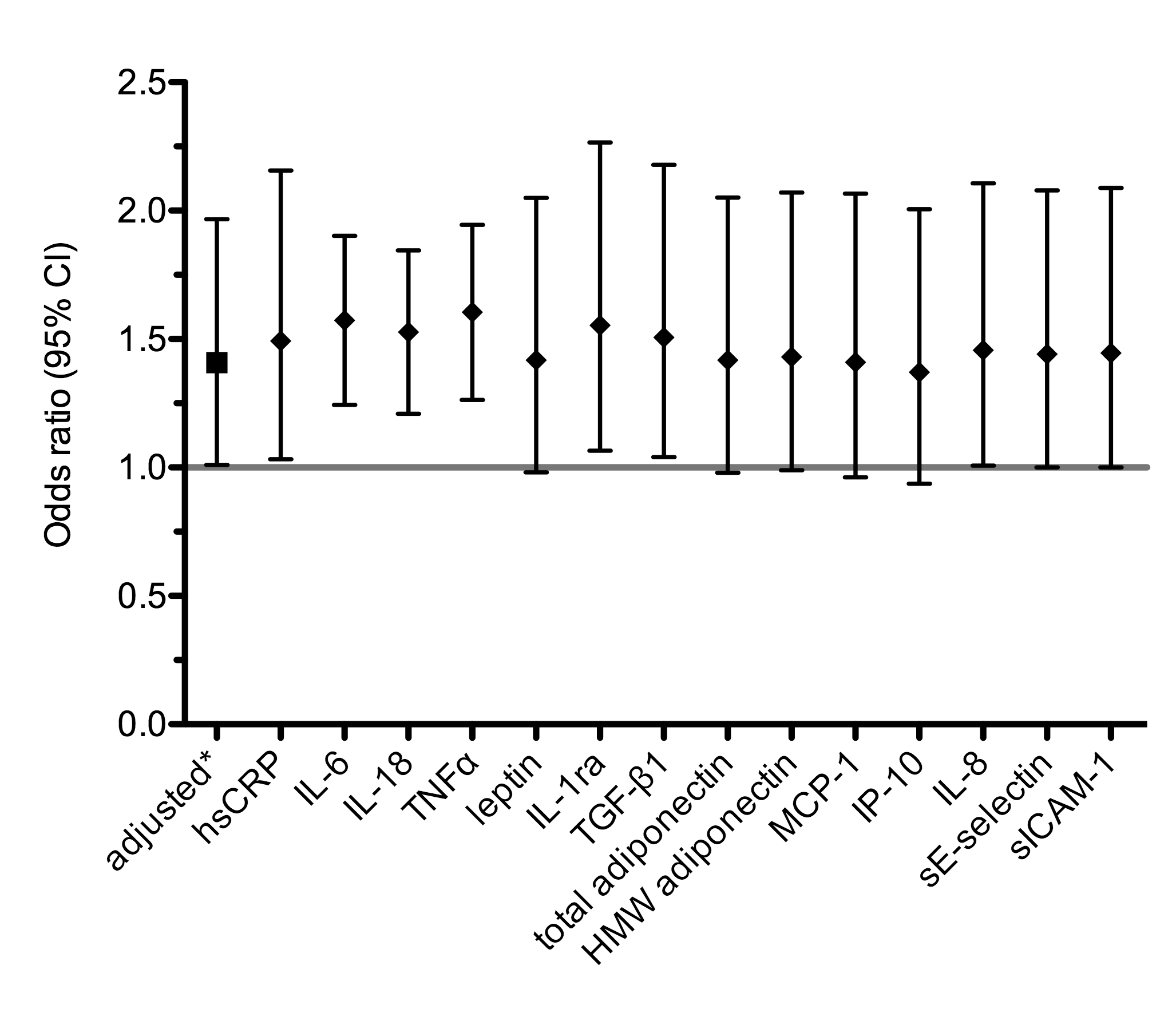

Supplement: Figure S1 — Impact of adjustment for immune mediators on the relationship between IGM and NOx†. *Adjusted for age, BMI, smoking status, education, exposure to indoor mould and season of blood sampling. All additional models are adjusted for the aforementioned covariables and the immune mediator indicated on the x-axis. (TIFF) [file pone.0083042.s001.tiff]

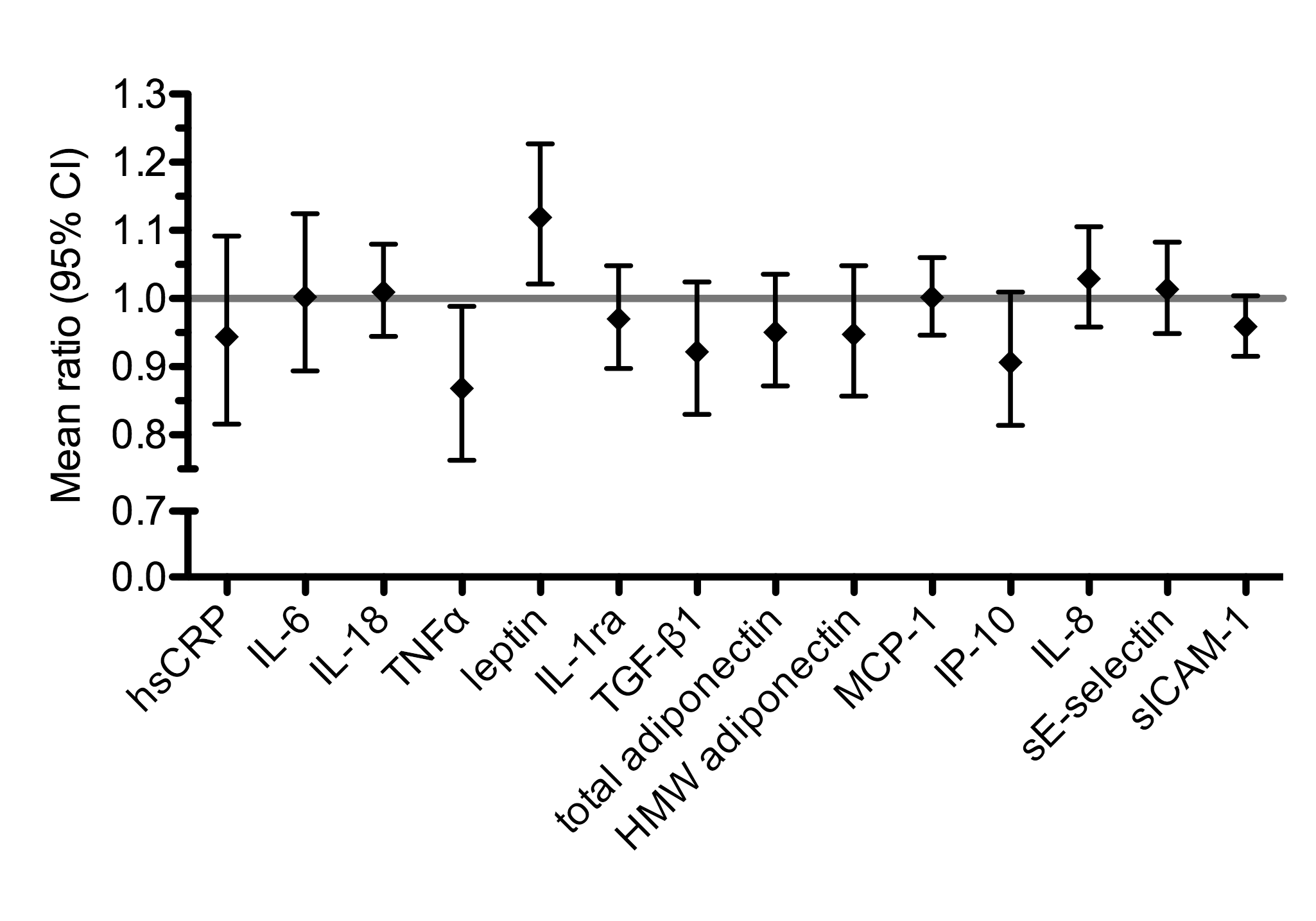

Supplement: Figure S2 — Association between circulating immune mediators and NOx†. Mean ratios are adjusted for age, BMI, smoking status, education, exposure to indoor mould and season of blood sampling. Ratios represent the relative increase of the particular serological marker concentration by 1-IQR increase of NOx † levels (IQR=43.16 µg/m3). (TIFF) [file pone.0083042.s002.tiff]
